# Engineered M2 macrophage-derived vesicles deliver DNase I for cfDNA clearance and multi-organ protection in sepsis

**DOI:** 10.1016/j.ijpx.2026.100528

**Published:** 2026-03-26

**Authors:** Fan Wu, Xinze Li, Xiayi Su, Zhangbin Tao, Zhiwei Huang, Zhongqiu Lu

**Affiliations:** a Department of Emergency, the First Affiliated Hospital of Wenzhou Medical University, Wenzhou 325035, China; b Wenzhou Key Laboratory of Emergency and Disaster Medicine, Wenzhou 325035, China; c Central Laboratory, Lishui Hospital of Wenzhou Medical University, The First Affiliated Hospital of Lishui University, Lishui People's Hospital, Lishui 323000, China; d Department of Pharmaceutics, School of Pharmaceutical Sciences, Wenzhou Medical University, Wenzhou 325035, China

**Keywords:** Sepsis, Cell-free DNA, DNase I, M2 macrophage–derived extracellular vesicles, Multi-organ injury

## Abstract

Sepsis is a life-threatening organ dysfunction caused by a dysregulated host response to infection. Despite advances in critical care, it remains a major global health challenge, highlighting the urgent need for novel therapeutic approaches. Elevated plasma cell-free DNA (cfDNA) has emerged as a critical driver of inflammation and tissue injury in sepsis. To neutralize its detrimental effects, DNase I can enzymatically degrade circulating cfDNA; however, the clinical application of native DNase I is limited by its rapid degradation and short circulation half-life. Here, we developed a bioengineered delivery system by encapsulating DNase I within M2 macrophage-derived extracellular vesicles (M2-EVs@DNase I), which possess intrinsic targeting ability, high biocompatibility, and low immunogenicity. In a cecal ligation and puncture (CLP)-induced sepsis model, administration of M2-EVs@DNase I significantly reduced circulating cfDNA levels by approximately 60% and suppressed TLR9 activation by about 49%. These effects were accompanied by a shift in macrophage polarization toward an anti-inflammatory M2 phenotype, reduced neutrophil activation, and significant attenuation of lung and kidney injury. Furthermore, treatment with M2-EVs@DNase I significantly improved biochemical indicators of organ function, including ALT, AST, UREA, and CRE levels. Together, these findings demonstrate that M2-EVs@DNase I effectively degrades pathogenic cfDNA and mitigates inflammatory responses, thereby protecting against sepsis-induced multi-organ injury. This study highlights the pathogenic significance of cfDNA in sepsis and introduces M2-EVs@DNase I as a promising biomimetic nanotherapeutic platform for sepsis treatment.

## Graphical abstract

**Figure:**
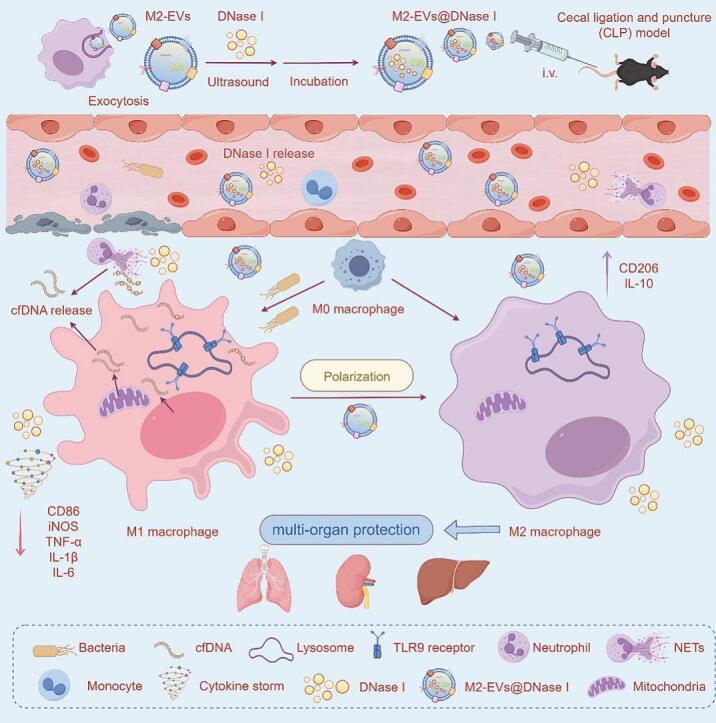

## 1 Introduction

Sepsis is a life-threatening condition characterized by organ dysfunction resulting from a dysregulated host response to infection (Singer et al., 2016; Gotts and Matthay, 2016). Despite significant progress in antimicrobial therapy and critical care, sepsis remains a leading cause of mortality worldwide, with an estimated 48.9 million cases and 11 million related deaths each year (Rudd et al., 2020). The high morbidity, mortality, and economic burden associated with sepsis underscore the urgent need for new therapeutic strategies that target its underlying pathophysiology rather than merely providing supportive care.

Recent research has revealed that circulating cell-free DNA (cfDNA) plays a pivotal role in the pathogenesis of sepsis (Li et al., 2026). cfDNA originates from dying cells through necrosis, apoptosis, and pyroptosis, as well as from neutrophil extracellular traps (NETs) released during inflammation (Stanley et al., 2024; Jiménez-Alcázar et al., 2017). Excessive accumulation of cfDNA amplifies systemic inflammation, tissue damage, and immune dysregulation (Li et al., 2025a). Elevated cfDNA levels have been detected in various inflammatory and autoimmune diseases, including sepsis, inflammatory bowel disease, and systemic lupus erythematosus (SLE) (Jacobs and Wong, 2016; Di Vincenzo et al., 2023; Han and Lo, 2021). Moreover, cfDNA quantification has shown promise as a biomarker for sepsis diagnosis and prognosis. Metagenomic next-generation sequencing (mNGS) of cfDNA from septic and non-septic patients enables comprehensive profiling of both microbial and host cfDNA components. Integrating cfDNA levels with microbial and host genomic signatures has led to diagnostic models that can predict 28-day mortality upon ICU admission (Jing et al., 2022). These findings highlight cfDNA not only as a biomarker but also as a pathogenic mediator and potential therapeutic target in sepsis.

In the innate immune system, antigen-presenting cells (APCs) recognize pathogenic and endogenous danger signals through pattern recognition receptors (PRRs), such as toll-like receptors (TLRs) (Murao et al., 2021; Kawasaki and Kawai, 2014). cfDNA functions as a potent damage-associated molecular pattern (DAMP) that binds and activates TLR9, a DNA-sensing receptor localized within endosomes of immune cells. This activation triggers NF-κB signaling, resulting in the transcription of pro-inflammatory cytokines and further amplification of systemic inflammation (Wang et al., 2020; van der Poll et al., 2017). Therefore, strategies aimed at degrading extracellular cfDNA and preventing TLR9 activation hold substantial potential for mitigating sepsis-induced inflammatory injury.

Deoxyribonuclease I (DNase I), a naturally occurring endonuclease that cleaves both single- and double-stranded DNA, has emerged as a promising candidate for cfDNA clearance (Laube et al., 1996). Clinically approved by the U.S. Food and Drug Administration (FDA) for cystic fibrosis, DNase I enzymatically digests extracellular DNA to reduce mucus viscosity and inflammatory load (Lauková et al., 2020). Beyond cystic fibrosis, accumulating evidence supports its broader therapeutic potential. DNase I administration alleviates inflammation in models of inflammatory bowel disease by reducing NETs accumulation and neutrophil infiltration (Wang et al., 2024). It also limits NETosis via inhibition of the cGAS–STING pathway, thereby ameliorating hemorrhagic transformation following tissue plasminogen activator (tPA) treatment in stroke (Wang et al., 2021). Our previous research demonstrated that DNase I treatment decreases cfDNA and NETs levels, reduces neutrophil recruitment, and prevents multi-organ dysfunction in murine sepsis models (Li et al., 2025b). However, the therapeutic efficacy of DNase I is severely restricted by its rapid degradation, poor stability, and short plasma half-life, necessitating the development of a delivery platform that enhances its pharmacokinetic properties (Davis Jr. et al., 1999; Penaloza Arias et al., 2022).

Extracellular vesicles (EVs) are nanoscale lipid bilayer structures secreted by nearly all cell types. They carry a diverse cargo of proteins, lipids, and nucleic acids, mediating intercellular communication and regulating immune responses (Welsh et al., 2024; de Jong et al., 2019; Kooijmans et al., 2021; Xu et al., 2025). Owing to their biocompatibility, low immunogenicity, and natural targeting ability, EVs have attracted substantial attention as drug delivery vehicles (Dave et al., 2025). Previous studies, including our own, have demonstrated that EV-based delivery systems can protect biologics from enzymatic degradation and improve their bioavailability. For instance, we reported that transferrin-modified milk-derived exosomes enhanced the oral stability of fibroblast growth factor 21 (FGF-21), thereby preserving its bioactivity in the gastrointestinal tract (Li et al., 2024). Particularly, EVs derived from M2 macrophages (M2-EVs) possess intrinsic anti-inflammatory and immunomodulatory properties, reflecting the phenotype of their parental cells. M2-EVs have been shown to alleviate acute lung injury by suppressing NF-κB/NLRP3 signaling through miR-709 transfer and to restore ovarian function by attenuating local inflammation in aged mice (Yang et al., 2024; Xiao et al., 2022). These findings suggest that M2-EVs not only serve as efficient delivery vehicles but also synergistically enhance therapeutic efficacy through their innate anti-inflammatory functions.

**Scheme 1:**
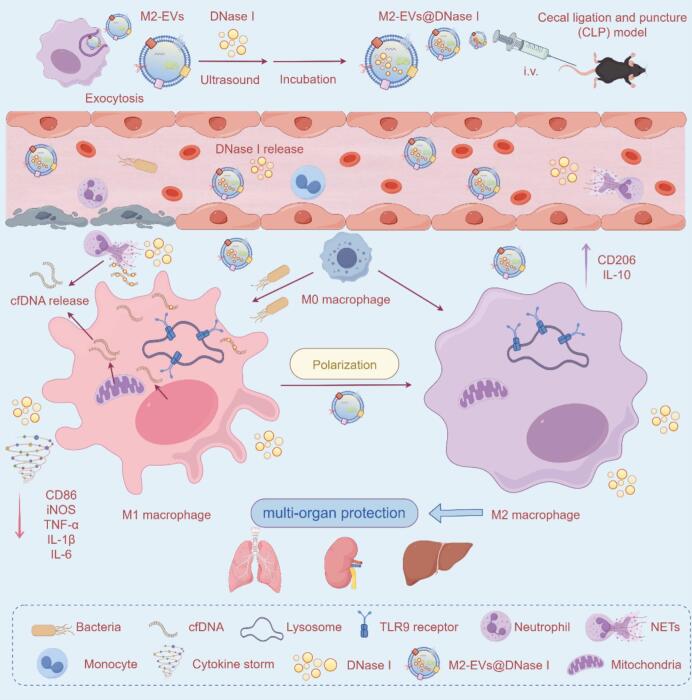
Preparation and therapeutic mechanism of M2-EVs@DNase I in sepsis. Following injection, M2-EVs@DNase I enter systemic circulation and gradually release the encapsulated DNase I. The released enzyme degrades excessive cfDNA, suppresses cytokine storms, and reprograms macrophages toward the anti-inflammatory M2 phenotype, thereby alleviating multi-organ injury and improving sepsis outcomes.

Based on these insights, we designed M2 macrophage-derived EVs as a biomimetic carrier for DNase I delivery to combat sepsis. In this study, we first confirmed a positive correlation between plasma cfDNA levels and disease severity in septic patients, emphasizing the pathogenic importance of cfDNA. Encapsulation of DNase I within M2-EVs (M2-EVs@DNase I) significantly improved its enzymatic stability, circulation time, and tissue biodistribution. More importantly, M2-EVs@DNase I treatment effectively degraded circulating cfDNA, suppressed TLR9-mediated inflammatory signaling, and promoted macrophage polarization toward an anti-inflammatory M2 phenotype (Scheme 1). Collectively, our findings reveal a previously unrecognized therapeutic mechanism centered on cfDNA clearance and introduce M2-EVs@DNase I as a promising and biocompatible nanotherapeutic platform for the treatment of sepsis.

## 2 Materials and methods

### 2.1 Materials

The Qubit dsDNA High Sensitivity Assay Kit (12640ES76) and DNase Viability Assay Kit (41322ES68) were bought from Yeasen (Shanghai, China). Cyanine5.5 (Cy5.5) and DNase I were acquired from MedChemExpress (MCE, NJ, USA). The Cell Counting Kit-8 (CCK-8, C40100) was bought from NCM Biotech (Suzhou, China). Red blood cell lysis buffer and hematoxylin and eosin (H&E) staining kit were purchased from Solarbio Science & Technology Co., Ltd. (Beijing, China). The One-Step TUNEL In Situ Apoptosis Kit (Green, Elab Fluor® 488) was bought from Elabscience® Biotechnology Co., Ltd. (Wuhan, China). Mouse tumor necrosis factor (TNF)-α (EM0010), interleukin (IL)-6 (EM0004), IL-1β (EM0029), and IL-10 (EM0005) enzyme-linked immunosorbent assay (ELISA) kits were obtained from HUABIO (Hangzhou, China).

### 2.2 Clinical study

A total of 78 patients diagnosed with sepsis between May 2024 and May 2025 in the Emergency Intensive Care Unit (EICU) were enrolled according to the Sepsis-3 International Consensus Definitions (Singer et al., 2016). Exclusion criteria included age < 18 years, malignancy, severe burns, pregnancy, or immunocompromised conditions (e.g., AIDS). Additionally, 26 healthy individuals were recruited as controls. This study was approved by the Ethics Committee in Clinical Research of the First Affiliated Hospital of Wenzhou Medical University (Approval No. KY2025-R323) and conducted in accordance with the Declaration of Helsinki. Whole blood was collected within 24 h after admission in EDTA-coated tubes and centrifuged at 1200*g* for 10 min at 4 °C. The supernatant was re-centrifuged at 14,000*g* for 10 min to remove residual debris. Plasma was aliquoted and stored at −80 °C. Comprehensive clinical data, including demographic information, laboratory results, and relevant clinical scores from the first day of admission, were collected for the cohort of sepsis patients and healthy controls. Baseline characteristics of all participants are summarized in Table S1.

### 2.3 Animal experiments

Male C57BL/6J mice (6—8 weeks) were obtained from Beijing Vital River Laboratory Animal Technology Co., Ltd. (Beijing, China). All animal procedures were approved by the Institutional Animal Care and Use Committee (IACUC) of the First Affiliated Hospital of Wenzhou Medical University (Approval No. WYYY-IACUC-AEC-2025-020). Mice were maintained under specific pathogen-free (SPF) conditions.

Sepsis was induced by cecal ligation and puncture (CLP) as previously described (Rittirsch et al., 2009; Huang et al., 2022). Briefly, after anesthesia and midline laparotomy, the cecum was ligated with 3–0 silk and punctured once with an 18-gauge needle. A small amount of fecal content was extruded before replacing the cecum into the peritoneal cavity. The abdominal wall was sutured, and mice were resuscitated with 5 mL/100 g sterile saline. Sham-operated mice underwent the same procedure without ligation or puncture. At 3 h post-CLP, mice received intravenous injections of DNase I or M2-EVs@DNase I (equivalent to 5 mg/kg DNase I). After 24 h, blood and major organs were harvested for analysis.

### 2.4 Quantification of cfDNA and MPO-DNA

Circulating cfDNA was extracted using the Serum/Plasma Circulating DNA Kit (DP339, TIANGEN) according to the manufacturer's instructions. Briefly, 100 μL plasma was lysed with Proteinase K and lysis buffer at 56 °C, followed by ethanol precipitation and purification using a spin column. After sequential washing steps, cfDNA was eluted in 100 μL elution buffer. The concentration of double-stranded DNA was determined using the Qubit dsDNA High-Sensitivity Assay Kit (12640ES76, Yeasen) according to the manufacturer's protocol. For quantification, samples or standards were mixed with the working detection reagent and incubated at room temperature for 2 min protected from light. Fluorescence signals were measured using a SpectraMax iD3 microplate reader (Molecular Devices) in fluorescence mode, and cfDNA concentrations were calculated based on the standard curve.

Plasma MPO–DNA complexes were quantified using a modified ELISA as previously described (Yang et al., 2020). Briefly, 96-well plates were coated overnight at 4 °C with anti-MPO monoclonal antibody (5 μg/mL; ab208670, Abcam) and blocked with 1% BSA. Plasma samples (20 μL) were incubated with peroxidase-conjugated anti-DNA monoclonal antibody (11,774,425,001, Cell Death Detection ELISA kit, Roche) for 2 h at room temperature with shaking. After washing, 100 μL peroxidase substrate was added and the reaction was incubated at 37 °C for 40 min. Absorbance was measured at 405 nm using a microplate reader.

### 2.5 RNA sequencing and transcriptome analysis

Whole blood samples from 31 septic patients and 10 healthy controls were collected for total RNA extraction using TRIzol® Reagent (15596018CN, Invitrogen). RNA sequencing was performed by Novogene Co., Ltd. (Beijing, China). Differentially expressed genes (DEGs) were identified using DESeq2 and edgeR with thresholds of |log_2_ fold change| ≥ 1 and *P* value <0.05. Overlapping DEGs identified by both methods were retained for downstream analyses. Hierarchical clustering was used to visualize transcriptional differences between sepsis patients and controls. Functional enrichment, including Gene Ontology (GO), Kyoto Encyclopedia of Genes and Genomes (KEGG), and Gene Set Enrichment Analysis (GSEA), was performed using the clusterProfiler R package.

### 2.6 Cell culture

Bone marrow-derived macrophages (BMDMs) were isolated as previously described (Huang et al., 2024). Bone marrow cells were cultured for 7 days in DMEM (Gibco, USA) supplemented with 10% fetal bovine serum (FBS, Gibco), 20 ng/mL macrophage colony-stimulating factor (M-CSF, PeproTech), and 1% penicillin–streptomycin. The culture medium was replaced twice during the induction period.

### 2.7 Isolation of M2 macrophage–derived extracellular vesicles (M2-EVs)

Mature M0 BMDMs were polarized into M2 macrophages by incubation with 20 ng/mL mouse IL-4 and IL-13 (PeproTech) for 24 h. Polarization efficiency was verified by flow cytometry using F4/80-FITC (2.5 μg/mL per million cells, 123107, Biolegend), CD86-APC (2.5 μg/mL per million cells, 105113, Biolegend) and CD206-PE (5 μg/mL per million cells, 141705, Biolegend). Immunofluorescence staining for F4/80 (1/50, ab204467, Abcam), iNOS (1/100, 53–5920-82, Invitrogen), and CD206 (1/300, ab64693, Abcam) further confirmed polarization.

After M2 induction, the medium was replaced with DMEM containing 10% exosome-depleted FBS (System Biosciences) and cultured for 24 h. The conditioned medium was sequentially centrifuged at 300*g* for 10 min, 2000 g for 20 min, and 10,000 g for 30 min to remove cells and debris. The supernatant was filtered through a 0.22 μm membrane (Corning, USA) and ultracentrifuged twice at 120,000 g for 90 min at 4 °C. The EV pellet was resuspended in PBS and stored at −80 °C. Protein concentration was determined by the BCA assay (Thermo Fisher Scientific).

### 2.8 Preparation of M2-EVs@DNase I

M2-EVs (0.5 mg/mL) and DNase I (5 mg/mL) were mixed at different mass ratios, sonicated, and incubated at 37 °C for 1 h. Unencapsulated DNase I was removed by ultrafiltration at 14,000 g for 10 min. Encapsulation efficiency (EE) and drug loading capacity (DLC) were calculated as follows:

$$\mathrm{EE}\%=\left(\text{Amount of DNase}\;I\;\text{in}\;M2-\mathrm{EVs}@\text{DNase}\;I\right)/\left(\text{Total amount of added DNase}\;I\right)\times 100$$

$$\mathrm{DLC}\%=\left(\text{Amount of DNase}\;I\;\text{in}\;M2-\mathrm{EVs}@\text{DNase}\;I\right)/\left(\text{Total amount of}\;M2-\mathrm{EVs}@\text{DNase}\;I\right)\times 100$$

### 2.9 Characterization of M2-EVs@DNase I

Particle size and zeta potential were analyzed using dynamic light scattering (DLS, Anton Paar, Austria), and particle concentration was measured by nanoparticle tracking analysis (ZetaView PMX 110, Particle Metrix, Germany). Morphology was examined via transmission electron microscopy (TEM, JEOL, Japan). The expression of exosomal markers CD9, CD63, and TSG101 was validated by Western blotting (all 1/1000). Protein profiles of M2-BMDM membranes, M2-EVs, M2-EVs@DNase I, and DNase I were compared by sodium dodecyl sulfate-polyacrylamide gel electrophoresis (SDS-PAGE) followed by Coomassie blue staining.

### 2.10 DNase I activity assay

DNase I activity was measured using a DNase Viability Assay Kit (41322ES68, Yeasen) according to the manufacturer's instructions. Briefly, samples were incubated with a fluorescence-labeled DNA substrate in reaction buffer, and fluorescence intensity was measured using a microplate reader in fluorescence mode. DNase activity was quantified based on a standard curve generated from DNase I standards. DNase I and M2-EVs@DNase I were incubated in PBS at 4 °C for 72 h to evaluate enzymatic stability, and DNase activity was measured at different time points. In addition, plasma DNase activity was determined in mice from different experimental groups.

### 2.11 Cytocompatibility assay

RAW264.7 cells and human umbilical vein endothelial cells (HUVECs, Procell) were seeded in 96-well plates and incubated with DNase I or M2-EVs@DNase I at concentrations equivalent to 0.1–1000 μg/mL DNase I for 24, 48, or 72 h. Cell viability was assessed using the CCK-8 assay, and absorbance was recorded at 450 nm using a SpectraMax iD3 microplate reader (Molecular Devices).

### 2.12 Hemocompatibility test

Mouse erythrocyte suspensions (2%) were incubated with DNase I or M2-EVs@DNase I (1 mg/mL DNase I equivalent) at 37 °C for 1 h. Double-distilled water and saline served as positive and negative controls, respectively. Then, all samples were centrifuged at 1000 rpm for 5 min. A 200 μL volume of the supernatant from each sample was used to determine the absorbance at 576 nm:

Hemolysis (%) = (OD_sample_ – OD_negative control_) / (OD_positive_ – OD_negative control_) × 100.

### 2.13 Biosafety study

To evaluate the long-term biosafety of the formulations, healthy C57BL/6J mice received intravenous injections of saline, DNase I, or M2-EVs@DNase I every other day for 7 days. At 24 h after the final injection, blood and major organs were collected. Hematological parameters, including red blood cell (RBC), hemoglobin (HGB), white blood cell (WBC), platelet (PLT), neutrophil (NEU), lymphocyte (LYMPH), and monocyte (MONO) were analyzed using an XN-1000 hematology analyzer (Sysmex, Japan). Serum biochemical markers, such as alanine aminotransferase (ALT), aspartate aminotransferase (AST), urea (UREA), and creatinine (CREZ) in serum were measured using an AU480 analyzer (Beckman Coulter, USA). Cytokine levels of TNF-α, IL-1β, and IL-6 were quantified by ELISA. Major organs were stained with H&E for histopathological evaluation.

### 2.14 Biodistribution of M2-EVs@DNase I

CLP-induced septic mice received intravenous injections of Cy5.5-labeled DNase I or M2-EVs@DNase I. Fluorescence imaging was performed at 3, 6, 12, and 24 h post-injection using AniView 100 (Guangzhou Biolight Biotechnology, China). In addition, DiI-labeled M2-EVs were intravenously injected into septic mice to investigate the pharmacokinetics of M2-EVs. In vivo fluorescence imaging was performed at 3, 6, 12, 24, and 48 h after administration. Major organs, including heart, liver, spleen, lung, and kidney were excised for ex vivo imaging.

### 2.15 Flow cytometry

Single-cell suspensions were prepared from peritoneal lavage fluid or bone marrow. After Fc receptor blocking with anti-CD16/32 (2.5 μg/mL per million cells, 156603, Biolegend), cells were stained with surface antibodies, including FITC anti-mouse F4/80 (123,107, Biolegend), PE/Cyanine7 anti-mouse/human CD11b (2.5 μg/mL per million cells, 101215, Biolegend), and APC anti-mouse CD86 (105,113, Biolegend). Cells were washed, fixed, and permeabilized to allow antibody access to intracellular targets. Subsequently, intracellular staining was performed using antibodies, including PE anti-mouse CD206 (141,705, Biolegend) and PE anti-mouse TLR9 (5 μg/mL per million cells, 159103, Biolegend). Data were acquired using a BD FACSCanto II cytometer (BD Biosciences, USA) and analyzed with FlowJo software.

### 2.16 Immunofluorescence (IF) and immunohistochemistry (IHC) staining

Following induction, cells were washed with phosphate-buffered saline (PBS) and fixed with 4% paraformaldehyde for 20 min, followed by permeabilization with 0.1% Triton X-100 for 15 min. Tissue samples were fixed in 4% paraformaldehyde for 24 h, rinsed thoroughly, dehydrated through a graded ethanol series, embedded in paraffin, and sectioned at a thickness of 5 μm. Sections were deparaffinized, rehydrated, and treated with 3% hydrogen peroxide for 10 min to block endogenous peroxidase activity, followed by antigen retrieval in citrate buffer at high temperature.

For IF staining, cells or tissue sections were blocked with 5% bovine serum albumin (BSA) at 37 °C for 30 min and incubated overnight at 4 °C with the following primary antibodies: Alexa Fluor® 647 anti-F4/80 (ab204467, Abcam), Alexa Fluor™ 488 anti-iNOS (53–5920-82, Invitrogen), anti-CD206 (ab64693, Abcam), and anti-KIM-1 (1/200, ab78494, Abcam). After rewarming and washing with PBS, samples were incubated with Alexa Fluor® 488-conjugated goat anti-rabbit IgG H&L secondary antibody (1/200, ab150077, Abcam) at 37 °C for 1 h. Nuclei were counterstained with Hoechst 33342 (P0133, Beyotime) or DAPI (S2110, Solarbio). Confocal images were acquired using a Nikon AX microscope (Nikon, Japan).

For IHC staining, paraffin-embedded lung tissue sections were blocked with 5% BSA and incubated overnight at 4 °C with primary antibodies against myeloperoxidase (MPO; 1/300, ab208670, Abcam) and TNF-α (1/50, sc-52,746, Santa Cruz). After washing with PBS, sections were incubated with HRP-conjugated goat anti-rabbit or anti-mouse IgG secondary antibodies. Signals were developed using a DAB substrate kit, and nuclei were counterstained with hematoxylin. Sections were then dehydrated, mounted with neutral resin, and visualized under a light microscope (Nikon Eclipse 80i, Japan).

Quantitative image analysis was performed using ImageJ software.

### 2.17 Statistical analysis

Statistical analyses were performed using SPSS 27.0, R 4.5.1, and GraphPad Prism 9. Data normality was assessed using the Shapiro–Wilk test prior to statistical comparisons.

For clinical data, normally distributed continuous variables are presented as mean ± standard deviation (SD) and compared using independent-sample *t*-tests. Non-normally distributed variables are expressed as median (interquartile range) and analyzed using the Mann–Whitney *U* test. Categorical variables were compared using the chi-square test. Correlations between cfDNA and clinical parameters were evaluated using Spearman correlation analysis. Receiver operating characteristic (ROC) curves were constructed to assess diagnostic performance, and the area under the curve (AUC) with 95% confidence intervals was calculated using the pROC package in R.

For experimental data, normally distributed datasets were analyzed using Student's *t*-test (two groups) or one-way ANOVA followed by Tukey's post hoc test (multiple groups). When normality was not satisfied, non-parametric tests (Mann–Whitney *U* test or Kruskal–Wallis test) were applied. Data are presented as mean ± SD. A two-tailed *P* < 0.05 was considered statistically significant.

Statistical significance was defined as *P < 0.05, **P < 0.01, ***P < 0.001, ****P < 0.0001.

## 3 Results and discussion

### 3.1 Plasma cfDNA levels positively correlated with sepsis severity

**Fig. 1:**
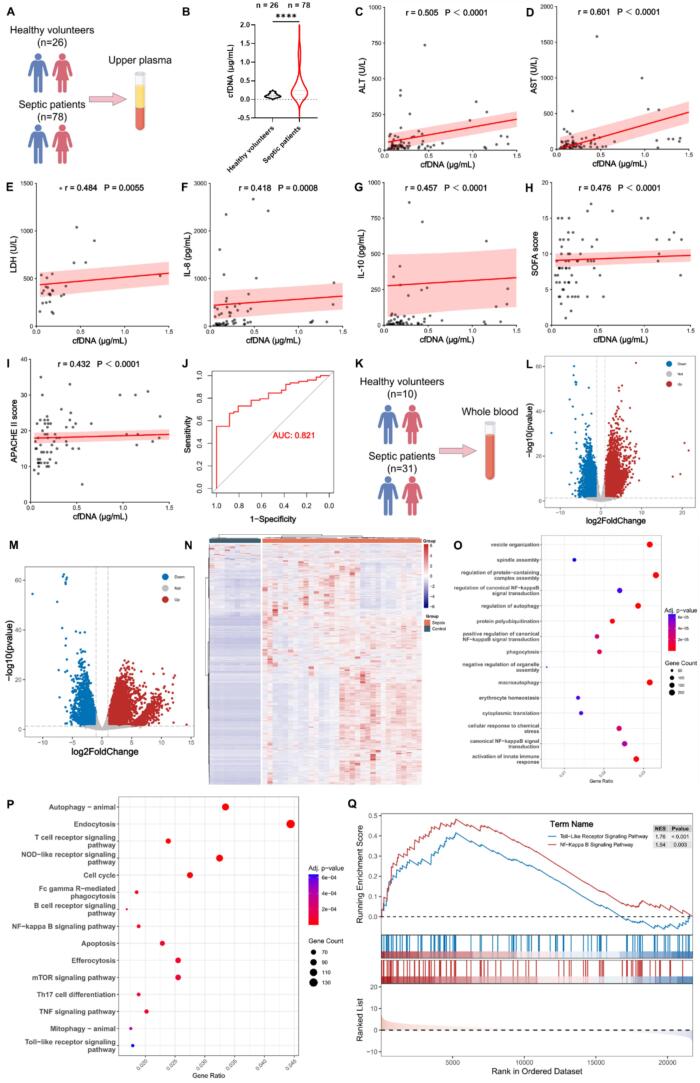
Elevated plasma cfDNA levels in septic patients and associated transcriptomic changes. (A) Comparison of plasma cfDNA levels between healthy controls (*n* = 26) and patients with sepsis (*n* = 78). (B) Quantification of plasma cfDNA in healthy individuals and septic patients. (C—I) Spearman correlation analysis between plasma cfDNA levels and (C) ALT, (D) AST, (E) LDH, (F) IL-8, (G) IL-10, (H) SOFA score, and (I) APACHE II score in septic patients. (J) ROC curve evaluating the diagnostic performance of plasma cfDNA in distinguishing sepsis patients from healthy controls. (K) Whole-blood transcriptome sequencing cohort, comprising healthy controls (*n* = 10) and septic patients (*n* = 31). (L, M) Volcano plots showing differential gene expression identified using DESeq2 and edgeR, respectively. (N) Hierarchical clustering heatmap of overlapping dDEGs distinguishing septic patients from healthy controls. (O) GO enrichment analysis of DEGs highlighting biological processes related to autophagy, innate immune activation, and NF-κB signaling. (P) KEGG pathway enrichment analysis demonstrating significant enrichment of immune- and inflammation-associated pathways. (Q) GSEA showing activation of Toll-like receptor and NF-κB signaling pathways in septic patients. ****P < 0.0001.

Previous studies have reported elevated circulating cfDNA levels in patients with sepsis (Jackson Chornenki et al., 2019). To validate these findings, we analyzed plasma cfDNA concentrations and clinical parameters from 78 patients with sepsis and 26 healthy controls (Fig. 1A). Our results showed that septic patients exhibited significantly higher neutrophil counts and plasma cfDNA levels compared with healthy individuals (Fig. S1A, Fig. 1B). To further investigate the clinical significance of cfDNA in sepsis, correlation analyses were performed between plasma cfDNA levels and key biochemical and clinical indicators. cfDNA levels exhibited strong positive correlations with key markers of liver and myocardial enzymes (ALT, AST, LDH), inflammatory cytokines (IL-8, IL-10), and disease severity (including SOFA and APACHE II scores) in septic patients, as shown in Fig. 1C-I (all *P* < 0.01). Receiver operating characteristic (ROC) curve analysis demonstrated that cfDNA effectively distinguished sepsis patients from healthy controls, with an area under the curve (AUC) of 0.821 (95% CI: 0.742–0.901, *P* < 0.0001) (Fig. 1J).

To elucidate the molecular mechanisms associated with cfDNA elevation in sepsis, whole-blood transcriptome sequencing was performed on samples from 31 septic patients and 10 healthy controls (Fig. 1K). Differential expression analyses using DESeq2 and edgeR identified 9222 genes (6433 upregulated, 2789 downregulated) and 10,187 genes (7432 upregulated, 2755 downregulated), respectively (Fig. 1L, M). Intersection of the two datasets yielded 8878 overlapping differentially expressed genes (DEGs), including 6296 upregulated and 2582 downregulated genes (Fig. S2A, B). Hierarchical clustering based on these DEGs revealed distinct transcriptional profiles that clearly separated septic patients from healthy controls (Fig. 1N). GO enrichment analysis demonstrated that these DEGs were significantly associated with key biological processes, including autophagy regulation, innate immune activation, and NF-κB signaling (e.g., canonical NF-κB pathway and its positive regulation) (Fig. 1O). KEGG analysis further indicated enrichment in several immune- and inflammation-related pathways, such as the T cell receptor, NOD-like receptor, B cell receptor, NF-κB, TNF, and Toll-like receptor signaling pathways, as well as apoptosis, efferocytosis, and Th17 cell differentiation (Fig. 1P). Consistently, GSEA revealed significant activation of the Toll-like receptor (NES = 1.76, *P* < 0.001) and NF-κB (NES = 1.54, *P* < 0.01) pathways, indicating a strong upregulation of innate immune and inflammatory responses in septic patients (Fig. 1Q).

Collectively, these findings demonstrate that plasma cfDNA levels are closely associated with sepsis severity and systemic inflammation. The transcriptomic evidence further highlights that excessive activation of innate immune and inflammatory pathways, particularly those mediated by Toll-like receptors and NF-κB signaling, plays a central role in the pathogenesis of sepsis.

### 3.2 Preparation and characterization of M2-EVs@DNase I

**Fig. 2:**
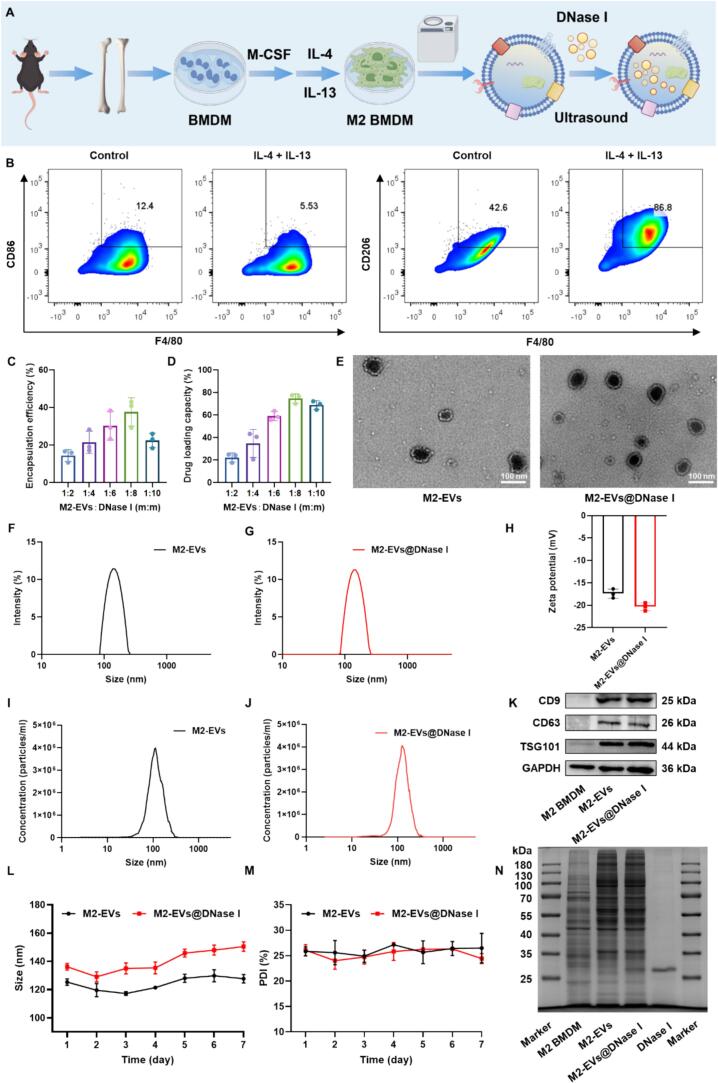
Preparation and characterization of M2-EVs@DNase I. (A) Schematic illustration of M2-EVs@DNase I preparation. (B) Flow cytometry analysis of BMDMs showing M1 macrophage (F4/80^+^CD86^+^) and M2 macrophage (F4/80^+^CD206^+^) with or without IL-4 and IL-13 treatment. *n* = 5. (C) EE and (D) DLC of M2-EVs@DNase I at different mass ratios. (E) TEM images of M2-EVs and M2-EVs@DNase I. Scale bar = 100 nm. (F, G) Hydrodynamic size distributions of M2-EVs and M2-EVs@DNase I. (H) Zeta potential of M2-EVs and M2-EVs@DNase I. (I, J) NTA of M2-EVs and M2-EVs@DNase I. (K) Representative Western blot showing expression of exosomal markers CD9, CD63, and TSG101 in M2 BMDMs, M2-EVs, and M2-EVs@DNase I. (L, M) Stability of M2-EVs and M2-EVs@DNase I over 7 days, as assessed by size and polydispersity index PDI. (N) SDS-PAGE analysis of M2 BMDMs, M2-EVs, M2-EVs@DNase I, and free DNase I. *n* = 3. Data are mean ± SD.

The preparation of M2-EVs@DNase I was illustrated in Fig. 2A. M2 macrophages were first induced from BMDMs, and successful polarization was confirmed. Flow cytometry analysis demonstrated that IL-4 and IL-13 stimulation significantly increased the proportion of F4/80^+^CD206^+^ cells to approximately 80%, whereas F4/80 and iNOS expression remained unchanged (Fig. 2B, Fig. S3A, B). Immunofluorescent staining further confirmed strong co-expression of F4/80 and CD206 in IL-4/IL-13-treated BMDMs (Fig. S3C–E). These findings collectively validated the successful induction of M2 macrophages. Subsequently, the cell culture supernatant was collected, and M2-EVs were isolated via ultracentrifugation.

M2-EVs@DNase I were prepared at various mass ratios using a sonication-incubation method. The EE% and DLC% were determined to be 37.71 ± 7.47% and 74.7 ± 4.1%, respectively, and an optimal DNase I-to-carrier mass ratio of 1:8 was selected (Fig. 2C, D). Transmission electron microscopy (TEM) revealed that both M2-EVs and M2-EVs@DNase I maintained characteristic saucer-shaped vesicular structures (Fig. 2E). The hydrodynamic diameter of M2-EVs measured 151.82 ± 3.33 nm, increasing slightly to 156.37 ± 5.20 nm after DNase I loading, while the zeta potential decreased from −17.41 ± 1.02 mV to −20.34 ± 0.88 mV (Fig. 2F–H). Nanoparticle tracking analysis (NTA) indicated particle concentrations of 8 × 10^10^ particles/mL for M2-EVs and 8.3 × 10^10^ particles/mL for M2-EVs@DNase I (Fig. 2I, J). Western blot analysis confirmed the presence of exosomal markers CD9, CD63, and TSG101 in both M2-EVs and M2-EVs@DNase I, whereas these markers were barely detectable in parental M2 macrophages (Fig. 2K). No significant changes in hydrodynamic size or polydispersity index (PDI) were observed for either formulation over seven days at 4 °C, indicating good stability (Fig. 2L, M). SDS-PAGE analysis revealed highly similar protein profiles for M2-EVs and M2-EVs@DNase I, with an additional band corresponding to DNase I in the latter, suggesting that vesicle integrity was maintained after drug loading (Fig. 2N). The enzymatic activities of DNase I and M2-EVs@DNase I were continuously monitored over 72 h. As shown in Fig. S4, the activity of free DNase I decreased to approximately 36% after incubation in PBS for 72 h, whereas M2-EVs@DNase I retained about 75% of its activity. These results indicate that encapsulation within M2-EVs significantly improves the stability of DNase I. Overall, these results demonstrate that M2-EVs@DNase I were successfully prepared, exhibiting preserved vesicular morphology, favorable physicochemical properties, and efficient DNase I encapsulation.

### 3.3 Biocompatibility and biodistribution of M2-EVs@DNase I

**Fig. 3:**
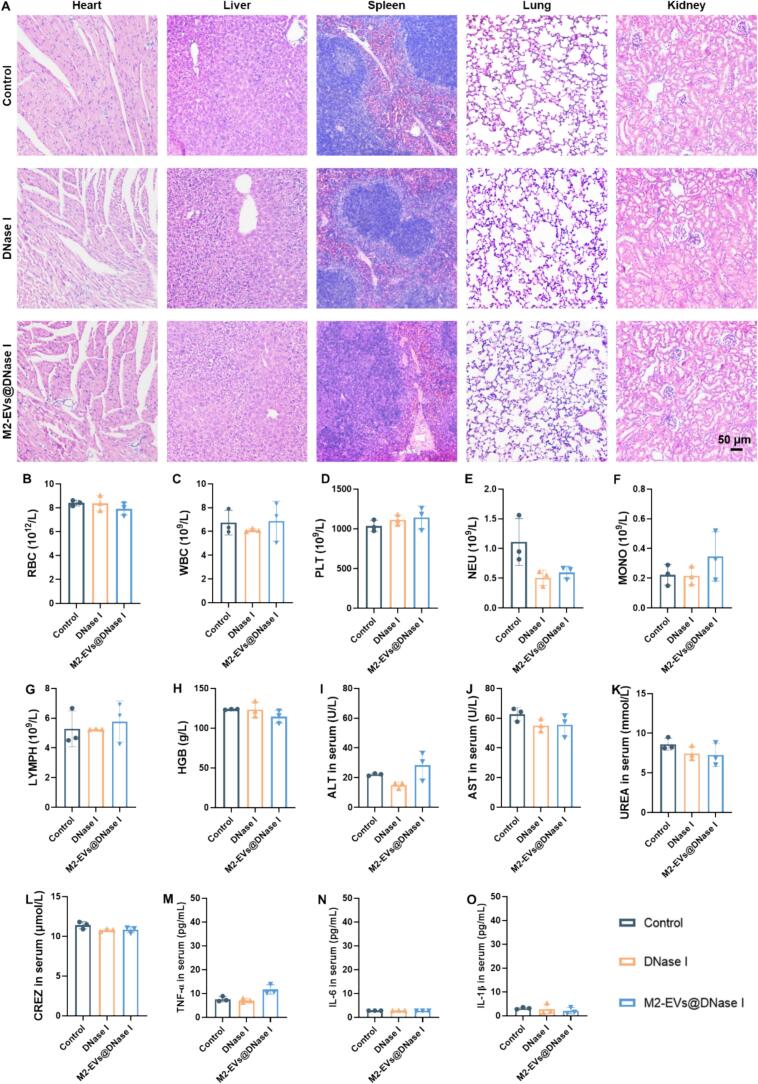
Biosafety assessment of DNase I and M2-EVs@DNase I. (A) Representative H&E staining images of the heart, liver, spleen, lung, and kidney from healthy mice after 7 days of treatment. Scale bar = 50 μm. (B—H) Hematological parameters including RBC, WBC, PLT, NEU, MONO, LYMPH, and HGB. (I-L) Serum biochemistry measurements of ALT, AST, UREA, and CREZ in each group. (M-O) Serum cytokine levels of TNF-α, IL-6, and IL-1β in each group. *n* = 3. Data are mean ± SD.

The cytotoxicity of DNase I and M2-EVs@DNase I (0.1–1000 μg/mL) was evaluated in RAW264.7 and HUVEC cells using a CCK-8 assay. Both formulations exhibited negligible cytotoxicity across the tested concentration range (Fig. S5A—F). Hemolysis assays using mouse erythrocytes showed minimal hemolysis in both groups, comparable to the negative control, confirming excellent hemocompatibility (Fig. S6A, B). To assess systemic safety in vivo, mice were intravenously administered DNase I or M2-EVs@DNase I. H&E staining of the heart, liver, spleen, lung, and kidney tissues revealed no pathological abnormalities in either treatment group compared with controls (Fig. 3A). Hematological analysis showed that RBC, WBC, PLT, NEU, MONO, LYMPH, and HGB levels remained within physiological ranges and were not significantly altered by treatment (Fig. 3B–H). Similarly, serum biochemical parameters, including ALT, AST, UREA, and CREZ, as well as inflammatory cytokines TNF-α, IL-6, and IL-1β, were all within normal limits and comparable to controls (Fig. 3I–O). Collectively, these results indicate that both DNase I and M2-EVs@DNase I possess excellent biocompatibility and systemic safety.

**Fig. 4:**
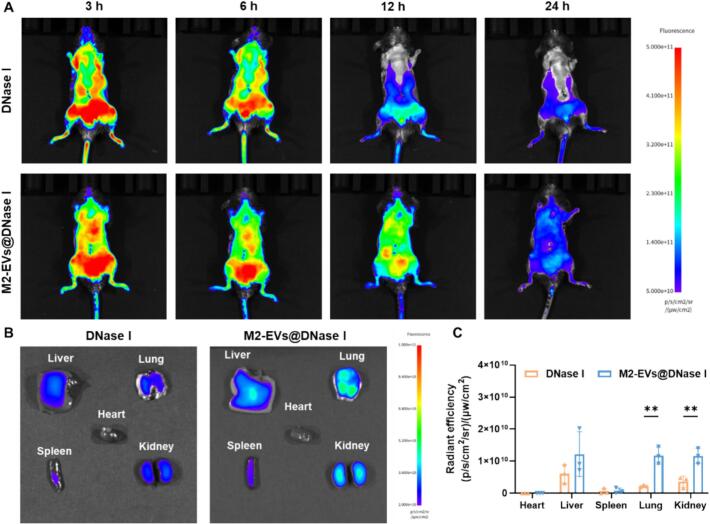
Biodistribution of M2-EVs@DNase I in septic mice. (A) Representative in vivo fluorescence images of septic mice following intravenous injection of Cy5.5-labeled DNase I or M2-EVs@DNase I at 3, 6, 12, and 24 h. (B) Ex vivo fluorescence images of major organs at 24 h post-administration. (C) Quantitative results of fluorescence intensity in major organs. n = 3. Data are mean ± SD. *P < 0.05, **P < 0.01.

The biodistribution of Cy5.5-labeled DNase I and M2-EVs@DNase I was subsequently examined in CLP-induced septic mice. Strong fluorescence signals were detected in both groups 3 h post-injection (Fig. 4A). The fluorescence in the DNase I group gradually declined and became nearly undetectable at 24 h, whereas M2-EVs@DNase I displayed sustained fluorescence, indicating prolonged circulation. Ex vivo imaging of major organs at 24 h post-injection showed significantly higher fluorescence intensity in the lungs and kidneys of the M2-EVs@DNase I group compared to DNase I alone (Fig. 4B, C). The preferential accumulation of M2-EVs@DNase I in the lungs and kidneys may be associated with increased vascular permeability and inflammatory cell infiltration in these organs during sepsis. In addition, EVs membrane proteins derived from parental M2 macrophages may contribute to tissue-specific interactions (Guan et al., 2025). In addition, the fluorescence signal of DiI-labeled M2-EVs was detectable at early time points and persisted up to 24 h after administration. The signal gradually decreased and was almost undetectable at 48 h, indicating that M2-EVs were largely cleared within this period (Fig. S7A). These findings suggest that M2-EVs encapsulation enhances the in vivo stability, biodistribution, and organ retention of DNase I, supporting its potential as a therapeutic platform for systemic inflammatory diseases.

### 3.4 M2-EVs@DNase I degraded cfDNA and modulated macrophage polarization

**Fig. 5:**
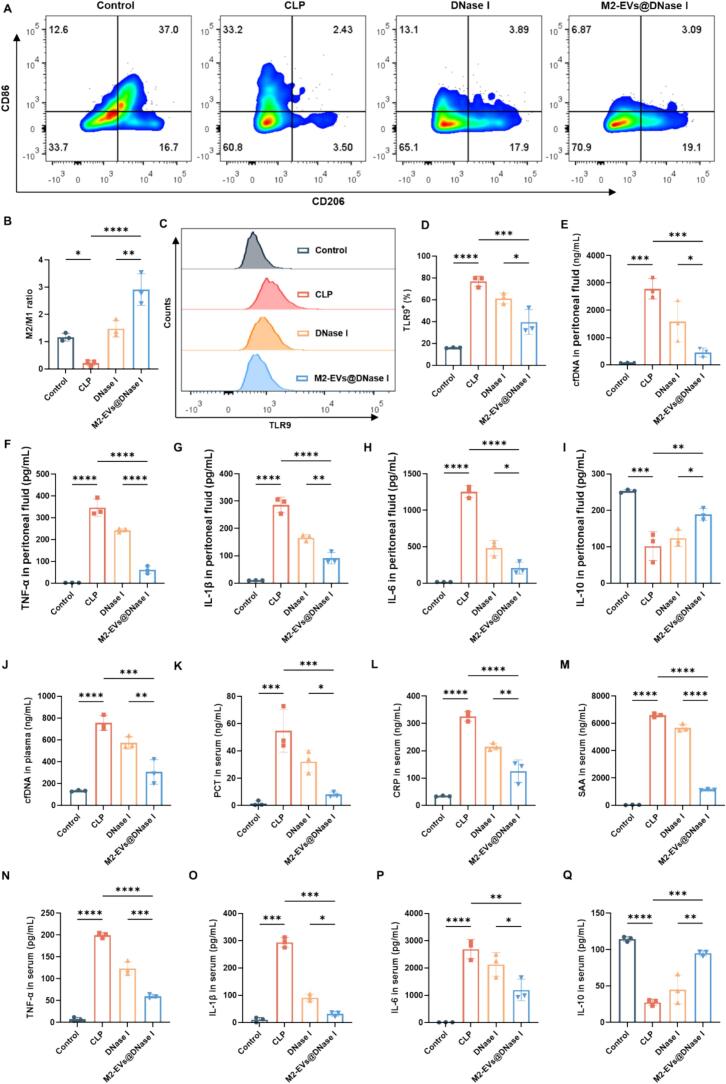
Therapeutic efficacy of M2-EVs@DNase I in septic mice. (A) Flow cytometry analysis of M1-phenotype macrophages (CD11b^+^F4/80^+^CD86^+^) and M2-phenotype macrophages (CD11b^+^F4/80^+^CD206^+^) in the peritoneal cavity. (B) Ratios of M2/M1 macrophages. (C, D) Flow cytometry and quantitative analysis of TLR9^+^ macrophages (CD11b^+^F4/80^+^TLR9^+^). (E) The levels of cfDNA in the peritoneal fluid. (F—I) The concentrations of TNF-α, IL-1β, IL-6, and IL-10 in the peritoneal fluid. (J) Plasma cfDNA levels. (K-Q) Serum levels of PCT, CRP, SAA, TNF-α, IL-1β, IL-6, and IL-10 in each group. *n* = 3. Data are mean ± SD. *P < 0.05, **P < 0.01, ***P < 0.001, ****P < 0.0001.

During sepsis, excessive circulating cfDNA could activate endosomal TLR9, triggering the NF-κB signaling cascade and driving macrophages toward a pro-inflammatory M1 phenotype. This process promotes the release of cytokines such as TNF-α, IL-6, and IL-1β, contributing to the systemic inflammatory response (Liu et al., 2014; Chen et al., 2022; Cui et al., 2025). The balance between M1 and M2 macrophage phenotypes is essential for regulating the initiation, progression, and resolution of sepsis; thus, modulating macrophage polarization represents a promising therapeutic strategy. To assess macrophage polarization during sepsis, peritoneal lavage fluid was collected 24 h after CLP surgery for flow cytometric analysis. Compared with the control group, CLP-induced sepsis markedly increased the proportion of pro-inflammatory M1 macrophages (F4/80^+^CD11b^+^CD86^+^) and decreased the proportion of anti-inflammatory M2 macrophages (F4/80^+^CD11b^+^CD206^+^), resulting in a significantly reduced M2/M1 ratio (Fig. 5A, B). Based on this baseline alteration, we next evaluated the immunomodulatory effects of the treatments. M2-EVs@DNase I administration substantially reversed the CLP-induced polarization imbalance by elevating the proportion of M2 macrophages while reducing the proportion of M1 macrophages, thereby restoring the M2/M1 ratio toward homeostatic levels (Fig. 5A, B). Furthermore, TLR9 expression was upregulated in peritoneal macrophages following CLP surgery but was significantly downregulated after M2-EVs@DNase I treatment (Fig. 5C, D). In addition, M2-EVs@DNase I effectively reduced cfDNA levels in the peritoneal lavage fluid (Fig. 5E). This treatment also decreased pro-inflammatory cytokines TNF-α, IL-1β, and IL-6 while enhancing the secretion of the anti-inflammatory cytokine IL-10 (Fig. 5F–I). Systemically, treatment with M2-EVs@DNase I significantly reduced plasma cfDNA and MPO–DNA levels as well as whole-blood neutrophil counts in septic mice, suggesting that it may suppress excessive NETs formation (Fig. 5J, Fig. S8A, B). Although increased NETs in the early stage of infection can facilitate pathogen trapping and killing, excessive NETs formation can act as damage-associated molecular patterns (DAMPs) and trigger uncontrolled inflammatory responses (Retter et al., 2025). Our previous findings showed that plasma cfDNA and MPO–DNA levels in septic mice increased markedly at approximately 3 h after CLP and remained elevated thereafter (Li et al., 2025b). Therefore, in the present study, treatment was administered at 3 h post-CLP to avoid premature degradation of NETs that may be required for early pathogen containment. At 24 h after CLP, plasma DNase I activity in septic mice tended to decrease compared with the control group, suggesting an impaired endogenous capacity to clear excessive cfDNA. After exogenous treatment with M2-EVs@DNase I, plasma DNase I activity was significantly increased (Fig. S9). During sepsis, excessive release of circulating cfDNA may overwhelm the clearance ability of endogenous DNase I (Cano-Gamez et al., 2025). In addition, cfDNA can activate TLR9 signaling and promote systemic inflammatory responses. The inflammatory milieu and oxidative stress present in sepsis may further compromise DNase I activity. The excessive production of cfDNA and insufficient clearance ability of DNase I during sepsis lead to sustained high levels of cfDNA (Zalghout and Martinod, 2025). Therefore, exogenous supplementation with M2-EVs@DNase I represents a potential strategy to enhance cfDNA clearance and mitigate sepsis-associated inflammation. Moreover, M2-EVs@DNase I also reduced serum biomarkers associated with inflammation and tissue injury, including PCT, CRP, SAA, TNF-α, IL-1β, and IL-6, while significantly increasing IL-10 levels (Fig. 5K-Q). These results demonstrate that M2-EVs@DNase I effectively cleared cfDNA, suppressed TLR9-mediated inflammatory signaling, and promoted macrophage polarization toward the M2 phenotype, thereby exerting potent anti-inflammatory and immunoregulatory effects in septic mice.

### 3.5 M2-EVs@DNase I prevented multiple organ dysfunction and improved therapeutic efficacy in sepsis

**Fig. 6:**
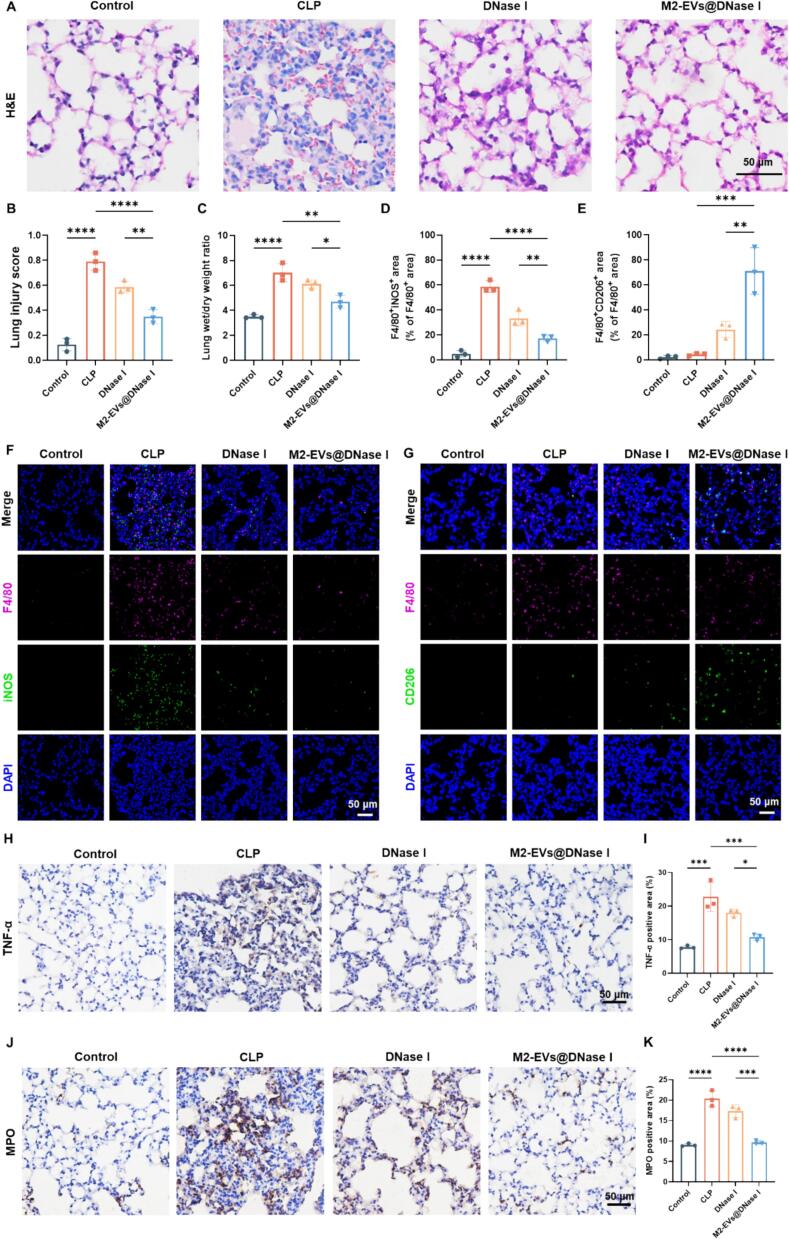
Protective effect of M2-EVs@DNase I against sepsis-induced lung injury. (A) Representative H&E staining images of lung tissues from each group. (B) Quantitative lung injury scores. (C) Lung wet/dry weight ratio of each group. (D, E) Quantification of M1 (F4/80^+^iNOS^+^) and M2 (F4/80^+^CD206^+^) macrophages, respectively. (F) Representative CLSM images of immunofluorescence staining for F4/80 (pink), iNOS (green), and DAPI (blue) of lung tissues in each group. (G) Representative CLSM images of immunofluorescence staining for F4/80 (pink), CD206 (green), and DAPI (blue) of lung tissues in each group. (H) Immunohistochemical staining for TNF-α in the lungs. (I) Quantification of TNF-α-positive cells. (J) Immunohistochemical images of MPO in the lungs. (K) Quantification of MPO-positive cells. Scale bar = 50 μm. *n* = 3. Data are mean ± SD. *P < 0.05, **P < 0.01, ***P < 0.001, ****P < 0.0001. (For interpretation of the references to colour in this figure legend, the reader is referred to the web version of this article.)

The lung is particularly vulnerable to sepsis-induced injury and represents a major target of cfDNA-mediated damage (Lefrançais et al., 2018). To evaluate the therapeutic efficacy of exogenously administered DNase I and its formulations, lung tissues from each group were examined. H&E staining revealed that septic mice exhibited obvious pathological alterations, including inflammatory cell infiltration, thickened alveolar septa, and disrupted alveolar architecture. Treatment with M2-EVs@DNase I effectively ameliorated these pathological changes, resulting in a significantly lower histopathological lung injury score (Fig. 6A, B). In addition, measurement of the lung wet/dry weight ratio indicated that M2-EVs@DNase I effectively reduced pulmonary edema in CLP-induced septic mice (Fig. 6C). During sepsis, alveolar macrophages play a central role in amplifying pulmonary inflammation by activating other immune cells and promoting the release of pro-inflammatory mediators, thereby disturbing local immune homeostasis (Wang and Wang, 2023; Jiao et al., 2021). To assess macrophage polarization within the lung microenvironment, immunofluorescence staining was performed. In the Control group, negligible expression of F4/80 (macrophage marker), iNOS (M1 marker), and CD206 (M2 marker) was observed, reflecting a resting macrophage state. In contrast, the CLP group showed a pronounced inflammatory response characterized by a substantial increase in F4/80^+^ macrophages and upregulated iNOS expression without a corresponding rise in CD206 levels. Notably, treatment with M2-EVs@DNase I markedly reduced iNOS expression and enhanced CD206 expression in F4/80^+^ macrophages, suggesting that it suppressed pro-inflammatory M1 polarization while promoting the anti-inflammatory M2 phenotype (Fig. 6D, E).

Furthermore, immunohistochemical analysis demonstrated elevated TNF-α and myeloperoxidase (MPO, a marker of neutrophil activation) expression in the CLP group (Fig. 6H, J). M2-EVs@DNase I treatment significantly decreased both TNF-α and MPO levels, indicating attenuation of cytokine release and neutrophil activation (Fig. 6I, K). Consistently, TUNEL staining demonstrated that M2-EVs@DNase I markedly reduced the number of apoptotic cells in lung tissue following CLP-induced sepsis (Fig. S10A, B). These results indicate that M2-EVs@DNase I confers robust protection against sepsis-induced lung injury by alleviating inflammatory damage, inhibiting neutrophil activation, reducing apoptosis, and reprogramming macrophage polarization toward an anti-inflammatory M2 phenotype.

**Fig. 7:**
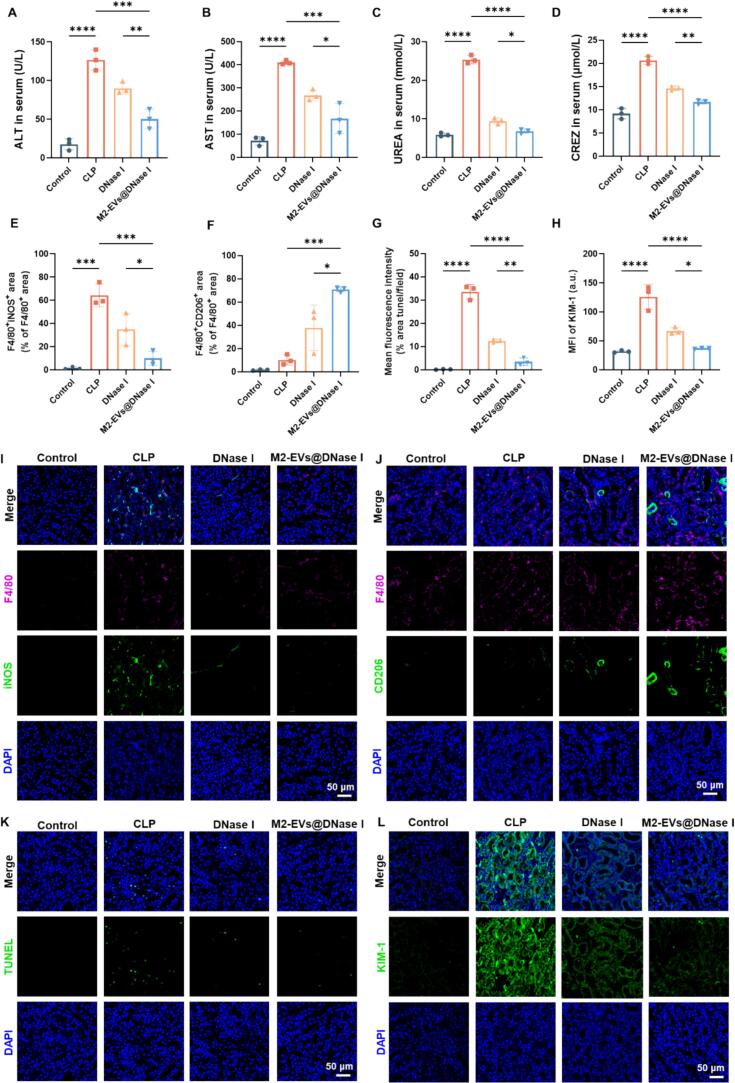
Therapeutic effects of M2-EVs@DNase I on sepsis-induced liver and kidney injury. (A-D) Serum levels of ALT, AST, UREA, and CREZ in each group. (E, F) Quantification of M1 (F4/80^+^iNOS^+^) and M2 (F4/80^+^CD206^+^) macrophages, respectively. (G) Quantification of TUNEL-positive cells. (H) Quantitative analysis of mean fluorescence intensity of KIM-1. (I) Representative CLSM images of immunofluorescence staining for F4/80 (pink), iNOS (green), and DAPI (blue) of kidney tissues from each group. (J) Representative CLSM images of immunofluorescence staining for F4/80 (pink), CD206 (green), and DAPI (blue) of kidney tissues from each group. (K) Representative TUNEL staining images (green) with DAPI nuclear counterstaining (blue). (L) Representative CLSM images of immunofluorescence staining for KIM-1 (green) and DAPI (blue) in the renal tissues. Scale bar = 50 μm. n = 3. Data are mean ± SD. *P < 0.05, **P < 0.01, ***P < 0.001, ****P < 0.0001. (For interpretation of the references to colour in this figure legend, the reader is referred to the web version of this article.)

Beyond pulmonary protection, M2-EVs@DNase I also mitigated systemic organ dysfunction. Treatment significantly reduced CLP-induced elevations in serum ALT, AST, UREA, and CREZ levels, indicating a protective effect against sepsis-induced hepatic and renal injury (Fig. 7A–D). Previous studies have shown that ALP serves as an endogenous detoxifying enzyme that mitigates the harmful effects of lipopolysaccharide (LPS) and plays an essential role in host defense and innate immunity (Pickkers et al., 2024; Su et al., 2006). In this study, ALP levels were significantly decreased in septic mice but were restored following M2-EVs@DNase I treatment, suggesting the recovery of innate detoxification capacity (Fig. S11A). Renal immunofluorescence analysis further revealed that CLP mice exhibited a substantial increase in F4/80^+^iNOS^+^ macrophages (M1 phenotype) and persistently low levels of F4/80^+^CD206^+^ macrophages (M2 phenotype) relative to controls (Fig. 7I, J). M2-EVs@DNase I treatment significantly reduced M1 macrophage infiltration and enhanced M2 macrophage abundance, confirming its ability to modulate macrophage polarization toward an anti-inflammatory state (Fig. 7E, F). Apoptotic cell death was assessed using the TUNEL assay, which detects fragmented DNA (Moore et al., 2021). The CLP group exhibited a significant increase in TUNEL-positive cells, indicating severe cellular apoptosis and DNA damage, whereas M2-EVs@DNase I treatment significantly reduced apoptotic cell numbers (Fig. 7G, K). In addition, kidney injury molecule-1 (KIM-1), a transmembrane glycoprotein specifically upregulated in renal proximal tubule epithelial cells following ischemic or toxic injury and correlated with sepsis severity, was significantly elevated in septic mice (Brozat et al., 2024). M2-EVs@DNase I treatment effectively decreased renal KIM-1 expression (Fig. 7H, L).

Collectively, these findings demonstrate that M2-EVs@DNase I exerts potent multi-organ protective effects in sepsis by modulating macrophage polarization, alleviating inflammatory injury, reducing apoptosis, and preserving hepatic and renal function.

## 4 Conclusion

In this study, we demonstrated a significant positive correlation between plasma cfDNA levels and sepsis severity in patients, underscoring cfDNA as a potential biomarker for disease progression. To therapeutically target cfDNA, we engineered M2 macrophage-derived extracellular vesicles as a delivery system for DNase I (M2-EVs@DNase I), aiming to prolong its systemic circulation and enhance its therapeutic efficacy in sepsis. The bioengineered M2-EVs@DNase I exhibited improved biodistribution, effectively degraded cfDNA, suppressed TLR9 activation, and reprogrammed macrophage polarization toward an anti-inflammatory M2 phenotype. Collectively, these effects mitigated the inflammatory response and alleviated sepsis-induced multi-organ injury. This study provides a promising biomimetic nanotherapeutic strategy for cfDNA clearance and immune modulation in the treatment of sepsis.

## CRediT authorship contribution statement

**Fan Wu:** Writing – original draft, Methodology, Investigation. **Xinze Li:** Validation, Investigation, Conceptualization. **Xiayi Su:** Methodology, Investigation. **Zhangbin Tao:** Data curation. **Zhiwei Huang:** Writing – review & editing, Funding acquisition, Conceptualization. **Zhongqiu Lu:** Supervision, Project administration, Funding acquisition.

## Declaration of competing interest

The authors declare that they have no known competing financial interests or personal relationships that could have appeared to influence the work reported in this paper.

## Data Availability

Data will be made available on request.

## Appendix A Supplementary data

Supplementary material Image 1
