# Supplementary material for: Engineered M2 macrophage-derived vesicles deliver DNase I for cfDNA clearance and multi-organ protection in sepsis
Source: Int J Pharm X. 2026 Mar 26;11:100528. doi: 10.1016/j.ijpx.2026.100528 (PMC13054405; doi:10.1016/j.ijpx.2026.100528)
Supplement: Supplementary file 1 — Supplementary material [file mmc1.docx]

**Supplementary Material**

**Engineered M2 macrophage-derived vesicles deliver DNase I for cfDNA clearance and multi-organ protection in sepsis**

Fan Wu^1,2#^, Xinze Li^1,2#^, Xiayi Su^1,2^, Zhangbin Tao^1,2^, Zhiwei Huang^3,4*^ and Zhongqiu Lu^1,2*^

^1^Department of Emergency, the First Affiliated Hospital of Wenzhou Medical University, Wenzhou 325035, China

^2^Wenzhou Key Laboratory of Emergency and Disaster Medicine, Wenzhou 325035, China

^3^Central Laboratory, Lishui Hospital of Wenzhou Medical University, the First Affiliated Hospital of Lishui University, Lishui People's Hospital, Lishui 323000, China

^4^Department of Pharmaceutics, School of Pharmaceutical Sciences, Wenzhou Medical University, Wenzhou 325035, China

^*^Corresponding authors: Zhiwei Huang (hzwpharm@163.com) and Zhongqiu Lu (lzhq@wmu.edu.cn)

^#^These authors contributed equally to this study.


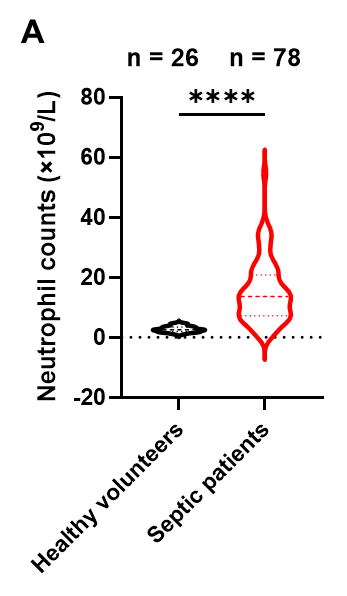


Figure S1. (A) Absolute neutrophil counts in patients with sepsis and healthy volunteers. *****P* < 0.0001.


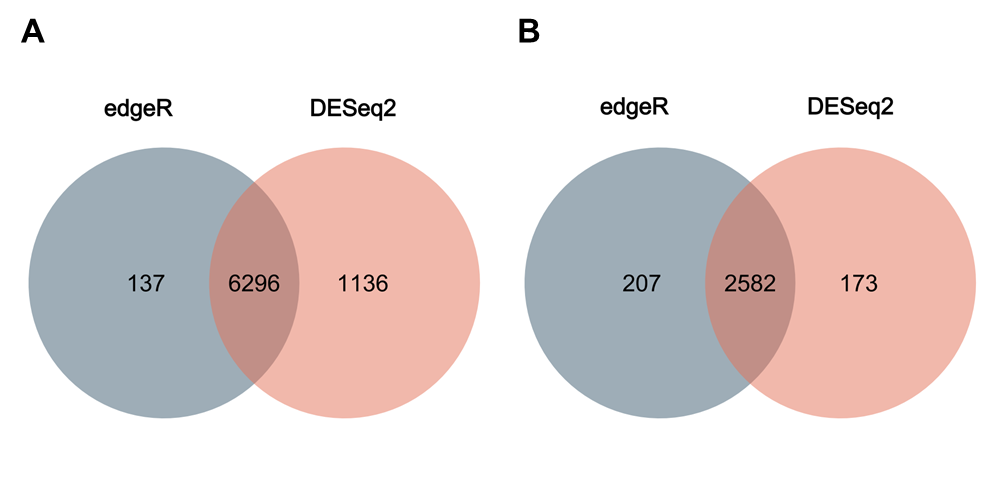
Figure S2. Venn diagrams showing the overlap of (A) upregulated and (B) downregulated differentially expressed genes (DEGs) identified by edgeR and DESeq2.


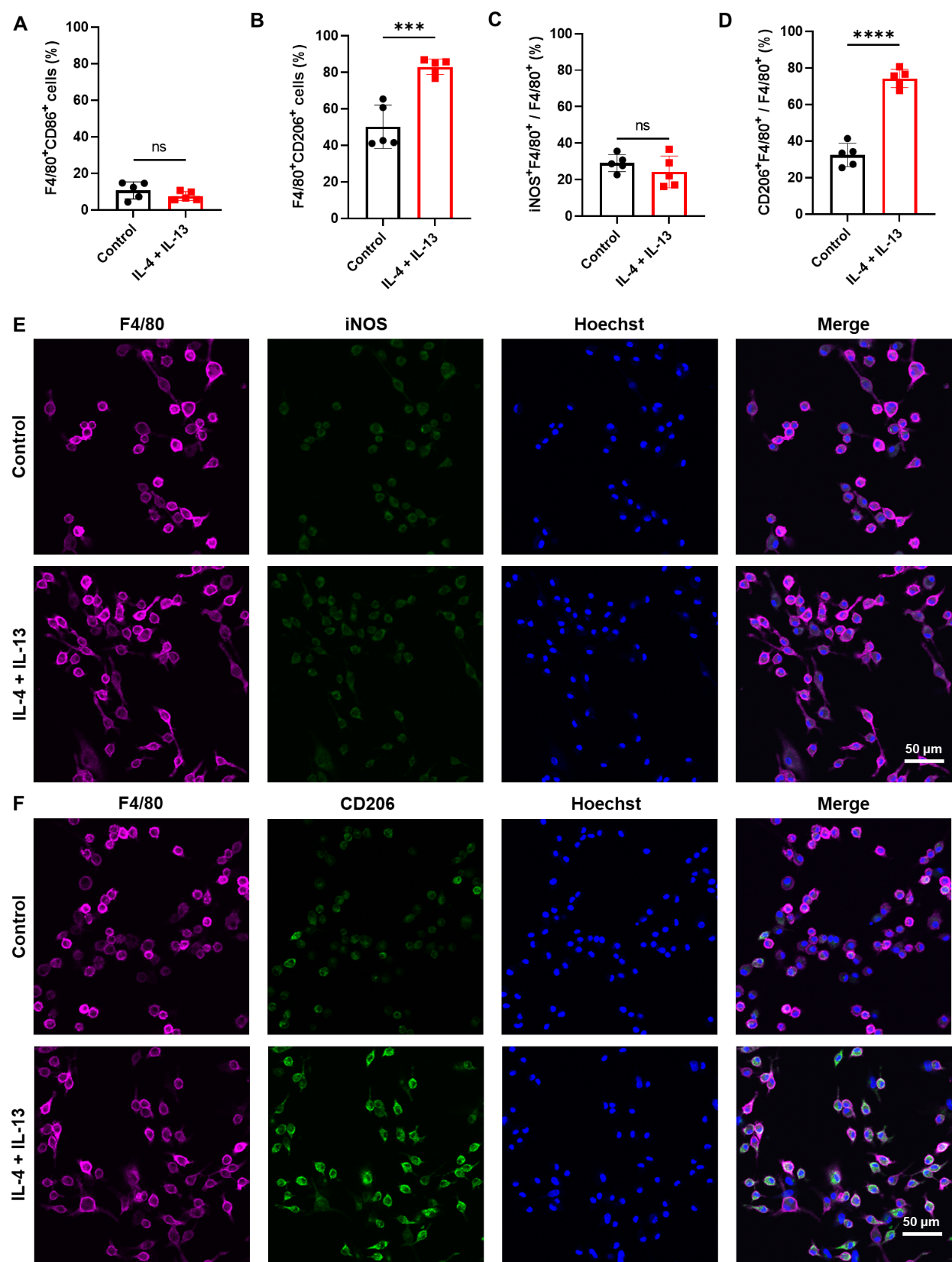


Figure S3. (A, B) Quantitative analysis of M1 macrophages (F4/80^+^CD86^+^) and M2 macrophages (F4/80^+^CD206^+^) among bone marrow–derived macrophages (BMDMs) with or without IL-4 and IL-13 treatment, as detected by flow cytometry (corresponding to Figure 2B). (C, D) Quantification of F4/80^+^iNOS^+^ and F4/80^+^CD206^+^ cells, respectively. (E, F) Representative immunofluorescence images of BMDMs stained for F4/80 (pink), iNOS/CD206 (green), and Hoechst (blue), with or without IL-4 and IL-13 treatment. Scale bar = 50 μm. n = 5. Data are mean ± SD. **P* < 0.05, ***P* < 0.01, ****P* < 0.001, *****P* < 0.0001. ns, not significant.


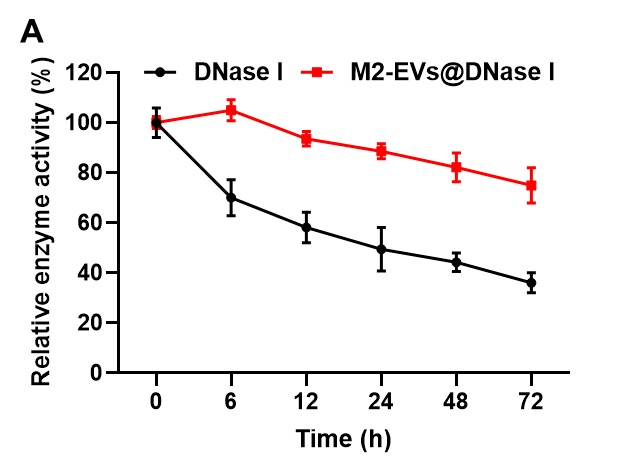


Figure S4. (A) Relative enzyme activity of DNase I and M2-EVs@DNase I after incubation in PBS at 4 °C for 72 h. n = 3. Data are mean ± SD.


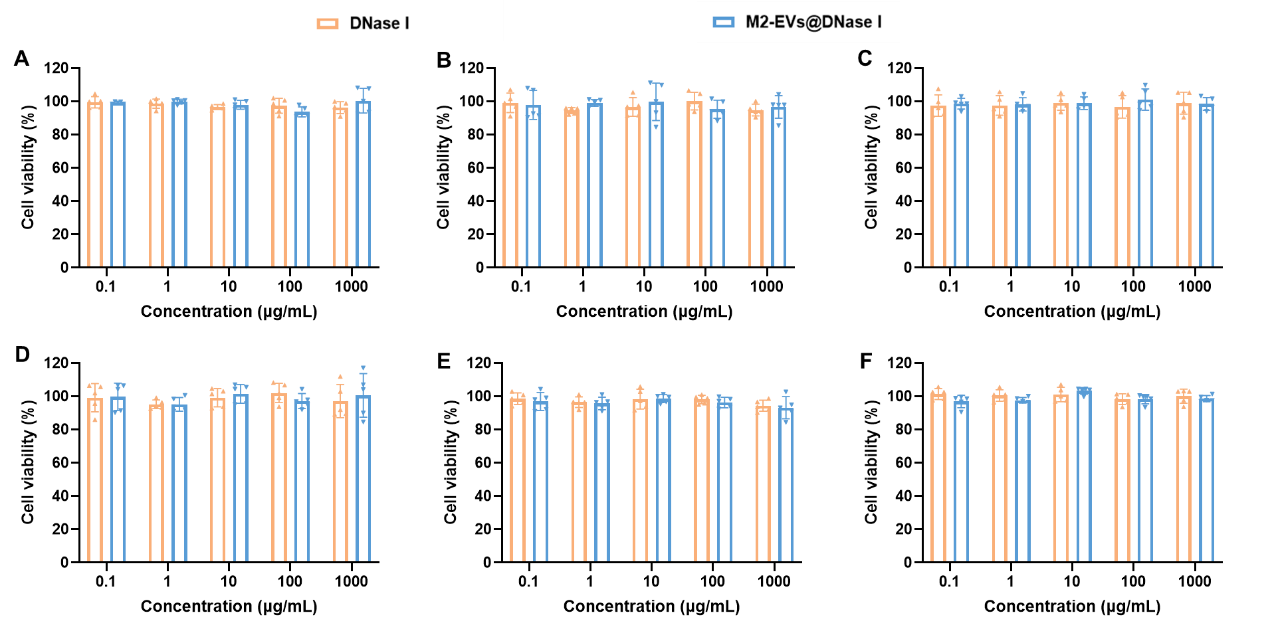


Figure S5. Cytotoxicity of DNase I and M2-EVs@DNase I. Cell viability of RAW264.7 macrophages treated with various concentrations of DNase I or M2-EVs@DNase I for (A) 24 h, (B) 48 h, and (C) 72 h. Cell viability of human umbilical vein endothelial cells (HUVECs) treated with DNase I or M2-EVs@DNase I at various concentrations for (D) 24 h, (E) 48 h, and (F) 72 h. n = 5. Data are mean ± SD.


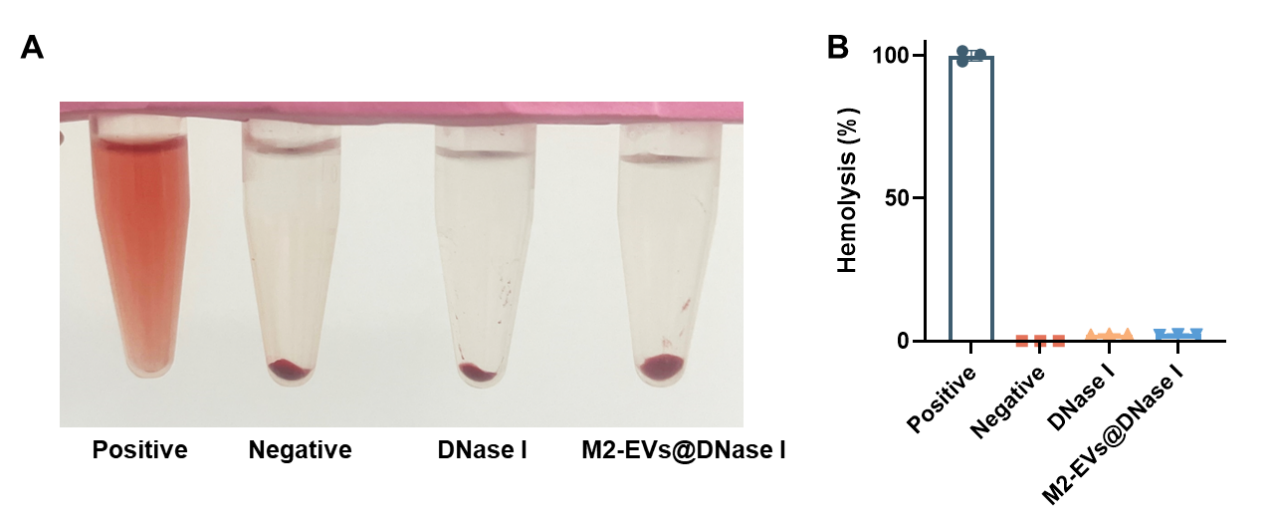


Figure S6. Hemolysis test. (A) Representative images and (B) hemolysis rates of samples treated with positive control (deionized water), negative control (0.9% NaCl), DNase I, and M2-EVs@DNase I. n = 3. Data are mean ± SD.


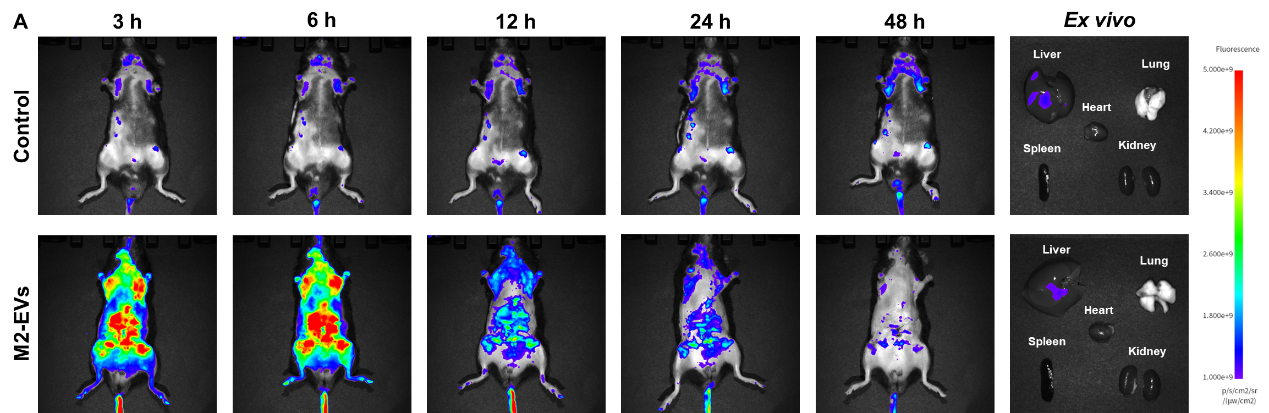


Figure S7. Biodistribution of M2-EVs. (A) *In vivo* fluorescence imaging was performed at 3, 6, 12, 24, and 48 h following intravenous injection of Dil-labeled M2-EVs. Major organs were collected for *ex vivo* fluorescence imaging at 48 h post-injection. n = 3.


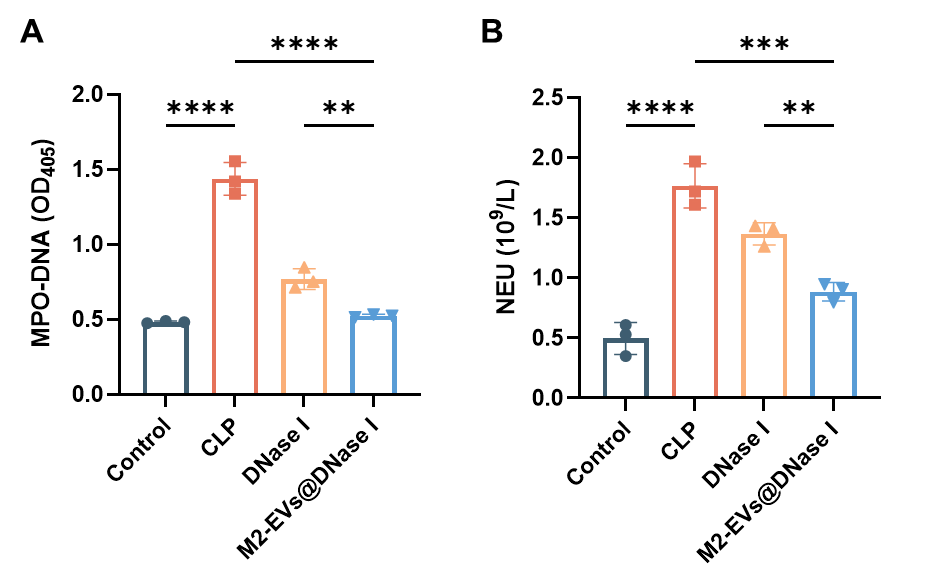


Figure S8. (A) Plasma MPO–DNA levels in mice from different groups. (B) Neutrophil counts in whole blood from different groups. n = 3. Data are mean ± SD. ***P* < 0.01, ****P* < 0.001, *****P* < 0.0001.


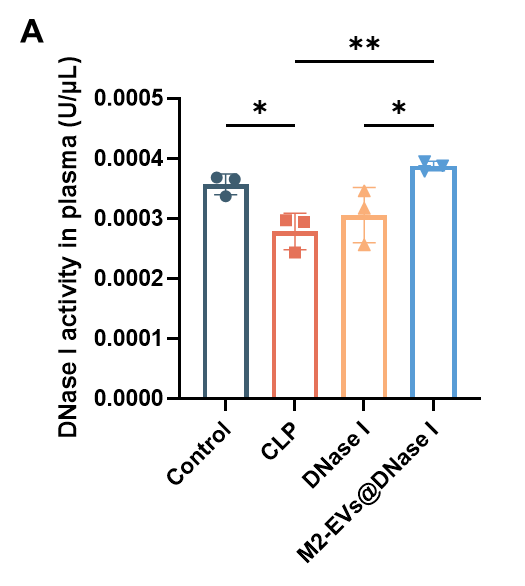


Figure S9. (A) Plasma DNase I activity in mice from different groups. n = 3. Data are mean ± SD. **P* < 0.05, ***P* < 0.01.


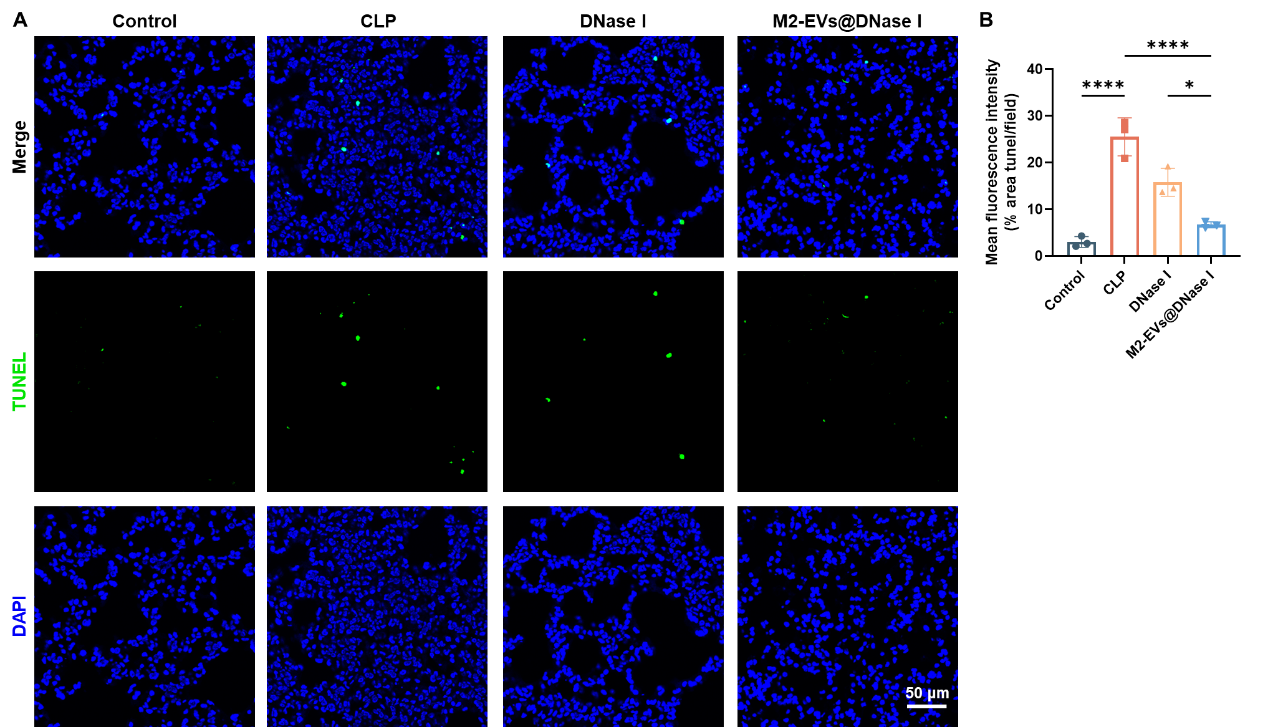


Figure S10. Apoptosis in renal tissues. (A) Representative images of TUNEL staining (green) and DAPI counterstaining (blue). (B) Quantitative analysis of TUNEL-positive cells. n = 3. Data are mean ± SD. **P* < 0.05, *****P* < 0.0001.


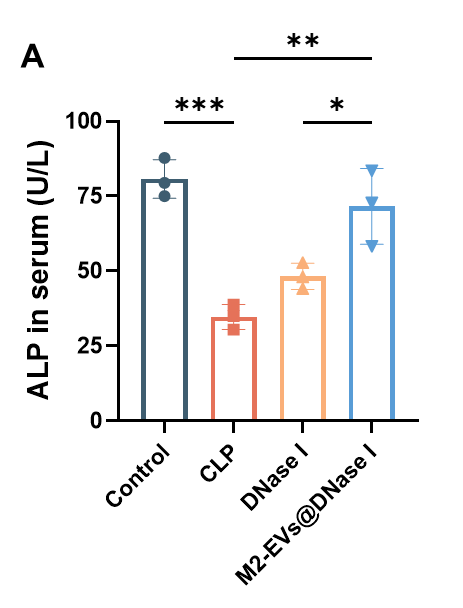


Figure S11. (A) Serum alkaline phosphatase (ALP) levels in each group. n = 3. Data are mean ± SD. **P* < 0.05, ***P* < 0.01, ****P* < 0.001.

**Table S1.** **Baseline characteristics of healthy volunteers and patients with sepsis.**

| **Characteristics** | **Septic patients**  **(n**$\boldsymbol{=}$**78)** | **Healthy volunteers**  **(n**$\boldsymbol{=}$**26)**^a)^ | ***P* value** |
| --- | --- | --- | --- |
| **Demographics** |  |  |  |
| Sex, male, n [%] | 46 (59.00%) | 13 (50.00%) | 0.496 |
| Age, median (IQR)^b)^, [years] | 65.5  (49.5-70.75) | 60  (58-61.75) | 0.142 |
| Outcome (Survival/Death) | 16/62 | 0/26 | 0.01* |
| Body mass index, median (IQR), [kg m^-2^] | 22.73  (20.05-26.1) | 23.11  (22.21-26.33) | 0.227 |
| **Laboratory data** |  |  |  |
| White blood cell count, median (IQR), [10^9^ L^-1^] | 15.33  (9.46-23.45) | 4.98  (4.12-5.95) | <0.001* |
| Neutrophil count, median (IQR), [10^9^ L^-1^] | 13.59  (7.27-20.6) | 2.58  (2.03-3.36) | <0.001* |
| Monocyte count, median (IQR), [10^9^ L^-1^] | 0.76  (0.48-1.17) | 0.3  (0.22-0.33) | <0.001* |
| Lymphocyte count, median (IQR), [10^9^ L^-1^] | 0.62  (0.42-1.08) | 1.86  (1.49-2.35) | <0.001* |
| Red blood cell count, mean$\text{±}$SD, [10^12^ L^-1^] | 3.54±0.89 | 4.75±0.56 | <0.001* |
| Platelet count, median (IQR), [10^9^ L^-1^] | 105  (52.25-171.25) | 195  (153-235.75) | <0.001* |
| Hemoglobin, median (IQR), [g L^-1^] | 106.5  (91-124) | 141.5  (128-154) | <0.001* |
| C-reactive protein, median (IQR), [mg L^-1^] | 182  (93.28-308.6) | 0.58  (0.5-0.84) | <0.001* |
| AST^c)^, median (IQR), [U L^-1^] | 83  (35.5-189.75) | 22  (20-25) | <0.001* |
| ALT^d)^, median (IQR), [U L^-1^] | 41  (22-114) | 19  (16-25) | <0.001* |
| Creatinine, median (IQR), [mmol L^-1^] | 149.5  (100.25-235.75) | 71  (60-80) | <0.001* |
| UREA, median (IQR), [mmol L^-1^] | 10.65  (7.43-16.75) | 5.6  (4.9-6) | <0.001* |
| Cystatin C, median (IQR), [mg L^-1^] | 1.89  (1.41-2.86) | 1.1  (1.04-1.15) | <0.001* |
| Creatine kinase, median (IQR), [U L^-1^] | 155.5  (48-604.75) | 166  (106-183) | 0.824 |
| SOFA score, median (IQR) | 9  (7-12) | NA^e)^ | NA |
| APACHE II score, median (IQR) | 18  (14-22.5) | NA^e)^ | NA |

^a)^n$=$sample size; ^b)^IQR, interquartile range; ^c)^AST, aspartate aminotransferase; ^d)^ALT, alanine transaminase; ^e)^NA, not applicable. Students’ *t*-test, and Chi-square test were used to calculate *P* values. *P* < 0.05 was considered statistically significant.
